# Laparoscopic low anterior resection in a patient with ventriculoperitoneal shunt: a case report

**DOI:** 10.1093/jscr/rjag084

**Published:** 2026-02-19

**Authors:** Salma Sait, Abdulaziz Saleem

**Affiliations:** Department of Surgery, International Medical Center, Hail Street, Al-Ruwais, PO Box 2172, Jeddah 21451, Saudi Arabia; Department of Surgery, Faculty of Medicine, King Abdulaziz University, Al Ehtifalat St, PO Box 80200, Jeddah 21589, Saudi Arabia

**Keywords:** hydrocephalus, ventriculoperitoneal shunt, laparoscopic surgery, low anterior resection, primary anastomosis, shunt safety

## Abstract

Hydrocephalus is commonly managed with a ventriculoperitoneal (VP) shunt, and the safety of laparoscopic surgery in these patients remains debated due to concerns regarding shunt malfunction and altered intracranial pressure. Evidence is mostly pediatric, with limited adult data. We report a case of laparoscopic low anterior resection (LAR) in an adult with a VP shunt. A 70-year-old man with a VP shunt placed 10 years prior was diagnosed with a well-differentiated rectosigmoid adenocarcinoma without metastasis. He underwent laparoscopic LAR using low insufflation pressure and intermittent desufflation. The shunt was left unclamped. The procedure was uneventful, and he was discharged on postoperative Day 4 with no neurological or surgical complications. Although historically considered high-risk, current evidence suggests that laparoscopic surgery can be safely performed in patients with VP shunts when careful perioperative measures are taken. Laparoscopic colon resection appears safe in selected VP shunt patients when appropriate precautions are implemented.

## Introduction

Hydrocephalus is a neurosurgical condition characterized by the accumulation of cerebrospinal fluid (CSF) in the cerebral ventricles that requires drainage to prevent the increase in intracranial pressure [1]. The ventriculoperitoneal (VP) shunt procedure has become the most common neurosurgical method for hydrocephalus [2]. There is still controversy regarding the safety of performing laparoscopic surgery in the presence of a VP shunt and potentially decreased cerebral compliance [3]. Most reported cases on the safety of laparoscopic surgery were in the pediatric population rather than adults. To our knowledge, this is the first case report of performing a low anterior resection (LAR) with primary anastomosis in the setting of a VP shunt.

## Case

**Figure 1:**
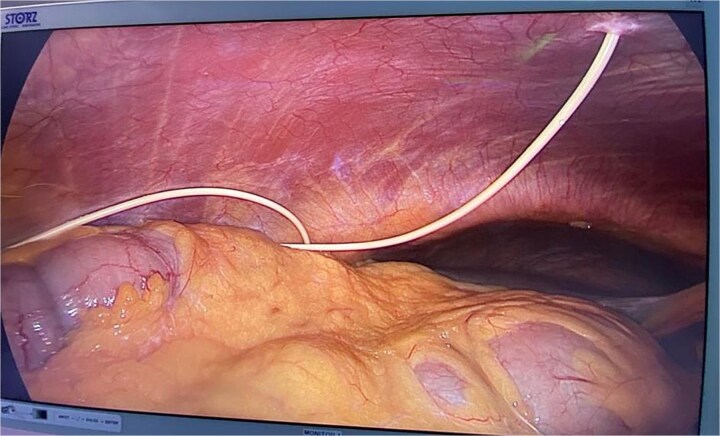
Picture demonstrating VP shunt in the peritoneal cavity.

A 70-year-old gentleman presented with bleeding per rectum. Initial evaluation revealed an upper rectal mass consistent with adenocarcinoma. His past medical history was significant for multiple comorbidities, including diabetes mellitus, hypertension, and hydrocephalus managed with a VP shunt inserted 10 years prior to presentation. Laboratory investigations showed anemia, prompting further evaluation. Colonoscopy demonstrated rectosigmoid lesions, and biopsy confirmed a well-differentiated adenocarcinoma. Staging workup revealed no evidence of metastatic disease. The patient was therefore scheduled for a laparoscopic LAR. Given the presence of the VP shunt, the neurosurgery team was consulted preoperatively due to concerns regarding the potential effects of pneumoperitoneum on shunt function. Based on their recommendations, mannitol was administered preoperatively along with standard prophylactic antibiotics. The shunt catheter was not clamped during the procedure. Intraoperatively, the VP shunt was visualized in the right upper quadrant (Fig. 1). The operation was performed with intermittent desufflation and at a low insufflation pressure. The patient underwent an uneventful laparoscopic LAR. Postoperatively, the course was smooth with no neurological complications. The patient was discharged on postoperative Day 4 and was subsequently followed up in the outpatient clinic, showing no neurological deficits or postoperative complications.

## Discussion

Laparoscopic surgery in patients with ventriculoperitoneal shunts (VPSs) was previously considered contraindicated due to the potential risk of shunt-related complications, including shunt malfunction secondary to increased intra-abdominal pressure, as well as catheter damage or infection. Some authors have reported that intracranial pressure may rise to as high as 25 mmHg when pneumoperitoneum pressure reaches 12 mmHg [1]. The safety of performing laparoscopic procedures in patients with VP shunts therefore remains a topic of ongoing debate, with some suggesting that pneumoperitoneum could impair cerebral compliance.

Earlier studies with limited monitoring of CSF shunt function—mainly based on clinical observation—have nevertheless reported laparoscopic procedures to be both safe and effective in this patient population [3]. Jackman *et al*. observed 19 laparoscopic operations performed in patients with VP shunts, using a mean insufflation pressure of 16 mmHg and an average operative time of 3 h; they found no clinically significant increase in intracranial pressure and recommended that routine anesthetic monitoring remain the standard of care [4].

In the pediatric population, Fraser *et al*. reported no episodes of air embolism and only one shunt infection in a series of 51 laparoscopic procedures in patients with VP shunts [5]. Similarly, Neale *et al*. conducted an *in vitro* study on nine shunts subjected to increased back pressure and observed no evidence of valve leakage under elevated pressure conditions [6].

Laparoscopic cholecystectomy without externalization of the shunt catheter has also been reported in adult patients with preexisting VP shunts, yielding favorable outcomes [7, 8]. Additionally, a previous report describing two cases of laparoscopic hemicolectomy demonstrated no perioperative complications or postoperative neurological deficits [9]. Barina *et al*. were the first to describe the clinical course of adults with VP shunts undergoing open appendectomy for appendicitis, similarly reporting no shunt-related complications such as malfunction or infection [10]. More recently, Monsellato *et al*. reported a successful robotic right hemicolectomy in a patient with a VPS, with a total pneumoperitoneum duration of 190 min [11].

In conclusion, laparoscopic colon resection in patients with VP shunts appears to be safe when appropriate precautions are taken, with no major intraoperative or postoperative complications reported.
